# *Isocitrate dehydrogenase 1* Gene Mutation Is Associated with Prognosis in Clinical Low-Grade Gliomas

**DOI:** 10.1371/journal.pone.0130872

**Published:** 2015-06-26

**Authors:** Ming-Yang Li, Yin-Yan Wang, Jin-Quan Cai, Chuan-Bao Zhang, Kuan-Yu Wang, Wen Cheng, Yan-Wei Liu, Wei Zhang, Tao Jiang

**Affiliations:** 1 Department of Neurosurgery, Beijing Tiantan Hospital, Capital Medical University, Beijing, China; 2 Beijing Neurosurgical Institute, Capital Medical University, Beijing, China; 3 Department of Neurosurgery, Second Affiliated Hospital of Harbin Medical University, Harbin, China; 4 Department of Neurosurgery, First Affiliated Hospital of Dalian Medical University, Dalian Medical University, Dalian, Liao Ning Province, China; 5 Department of Neurosurgery, the First Hospital of China Medical University, Shenyang, China; 6 China National Clinical Research Center for Neurological Diseases, Beijing, China; 7 Center of Brain Tumor, Beijing Institute for Brain Disorders, Beijing, China; University of North Carolina School of Medicine, UNITED STATES

## Abstract

*Isocitrate dehydrogenase 1* gene mutations are found in most World Health Organization grade II and III gliomas and secondary glioblastomas. *Isocitrate dehydrogenase 1* mutations are known to have prognostic value in high-grade gliomas. However, their prognostic significance in low-grade gliomas remains controversial. We determined the predictive and prognostic value of *isocitrate dehydrogenase 1* status in low-grade gliomas. The association of *isocitrate dehydrogenase 1* status with clinicopathological and genetic factors was also evaluated. Clinical information and genetic data including *isocitrate dehydrogenase 1* mutation, *O 6-methylguanine DNA methyltransferase* promoter methylation, 1p/19q chromosome loss, and *TP53* mutation of 417 low-grade gliomas were collected from the Chinese Glioma Genome Atlas database. Kaplan–Meier and Cox proportional hazards regression analyses were performed to evaluate the prognostic effect of clinical characteristics and molecular biomarkers. *Isocitrate dehydrogenase 1* mutation was identified as an independent prognostic factor for overall, but not progression-free, survival. Notably, *isocitrate dehydrogenase 1* mutation was found to be a significant prognostic factor in patients with oligodendrogliomas, but not in patients with astrocytomas. Furthermore, *O 6-methylguanine DNA methyltransferase* promoter methylation (p = 0.017) and *TP53* mutation (p < 0.001), but not 1p/19q loss (p = 0.834), occurred at a higher frequency in *isocitrate dehydrogenase 1*-mutated tumors than in *isocitrate dehydrogenase 1* wild-type tumors. Younger patient age (p = 0.041) and frontal lobe location (p = 0.010) were significantly correlated with *isocitrate dehydrogenase 1* mutation. Chemotherapy did not provide a survival benefit in patients with *isocitrate dehydrogenase 1*-mutated tumors. *Isocitrate dehydrogenase 1* mutation was an independent prognostic factor in low-grade gliomas, whereas it showed no predictive value for chemotherapy response. *Isocitrate dehydrogenase 1* mutation was highly associated with *O 6-methylguanine DNA methyltransferase* promoter methylation and *TP53* mutation.

## Introduction

Low-grade gliomas (LGGs) are the most common primary brain tumors and are comprised of specific histological subtypes, including astrocytomas, oligodendrogliomas, and oligoastrocytomas. LGGs have been classified as grade II tumors based on the histopathological and clinical criteria established by the World Health Organization (WHO) [1]. Although histopathology has been considered the gold standard for the pathological classification of brain tumors, it has become increasingly clear that these criteria still have limitations. Prognosis has been shown to vary widely in patients with the same histologic subtype, which may due to the significant genetic variation among brain tumors. Therefore, identification of molecular characteristics is essential for tumor diagnosis and outcome prediction. Low-grade diffuse gliomas (WHO grade II) are well-differentiated and slow-growing tumors that diffusely infiltrate surrounding brain structures. However, these tumors show a consistent tendency to recur even after surgical resection. As the management of patients with LGGs remains controversial, the development of markers that unfailingly predict tumor performance would be beneficial in treatment planning.

Recent genome-wide mutational analyses have demonstrated the presence of i*socitrate dehydrogenase 1* (*IDH1*) mutations in more than 70% of WHO grade II and III astrocytomas, oligodendrogliomas, and secondary glioblastomas (GBMs) [2, 3], whereas fewer than 5% of primary GBMs harbor this mutation [4]. In addition, accumulating research has confirmed that *IDH1* mutation occurs early in gliomagenesis [5], which suggests that this genetic event drives tumor progression. Therefore, *IDH1* status is a useful biomarker in assisting molecular-based classification [6]. However, to date, the usefulness of *IDH1* mutation as a predictive marker for treatment response and survival outcome in patients with LGGs is still unknown. Studies investigating the role of *IDH1* mutation in predicting chemotherapy response in LGGs have produced conflicting results. A few studies have suggested that *IDH1* mutation is associated with better outcome and sensitivity to temozolomide; however, evidence of its predictive value for response to alkylating agents, 1-(2-chloroethyl)-3-cyclohexyl-L-nitrosourea, and vincristine chemotherapy is lacking [7–9].

Many previous studies have suggested that the prevalence of *IDH1* mutations is particularly high in LGGs with 1p/19q deletion [10], *TP53* mutation [5], and O *6*-methylguanine-DNA *m*ethyltransferase (*MGMT*) gene promoter methylation [11], which has been shown to play a role in predicting survival in patients with newly diagnosed GBM [11]. However, the clinical and histopathological characteristics associated with *IDH1* status in LGGs have not yet been systematically elucidated.

Our previous study demonstrated that *IDH1* mutation was a prognostic factor and correlated with various clinicopathological parameters in primary GBM [12]. In the present study, we focused on the predictive and prognostic value of *IDH1* mutations for survival and treatment response in a large series of patients with LGGs (n = 417). We also examined the association of *IDH1* status with clinicopathological parameters including age, gender, tumor location, histology, *MGMT* promoter methylation, *TP53* mutation, and 1p/19q chromosome deletion.

## Materials and Methods

### Study cohort

A total of 417 clinical patients with LGGs from the Chinese Glioma Genome Atlas (CGGA) database were analyzed. All tumors were pathologically diagnosed as WHO grade II gliomas. Clinical information including patient gender, age at the time of diagnosis, tumor location, preoperative Karnofsky Performance Status (KPS) score, extent of resection, adjuvant chemotherapy and radiotherapy, and the recorded date of disease progression or death were systematically reviewed. This study was approved by the Ethics Committee of Beijing Tiantan Hospital, and written informed consent was obtained from all patients included in this study.

### DNA extraction

Materials were selected for DNA extraction after careful examination of corresponding hematoxylin and eosin-stained sections. All selected samples contained at least 80% of vital tumor. Genomic DNA was extracted from frozen tumor tissues using the QIAamp DNA Mini Kit (Qiagen) according to the manufacturer’s protocol. RNA sequencing was performed as described in our previous study [12].

### DNA pyrosequencing for *IDH1* mutation

The genomic region spanning wild-type R132 of *IDH1* was analyzed by pyrophosphate sequencing using the following primers: (forward) 5'-GCTTGTGAGTGGATGGGTAAAAC-3' and (reverse) 5'-biotin-TTGCCAACATGACTTACTTGATC-3'. Polymerase chain reaction (PCR) was performed using the ABI PCR System 9700 (Applied Biosystems). Polymerase chain reaction amplification was performed in a 40 μl reaction volume containing 1 μl each of forward and reverse primer (10 μM), 4 μl 10× buffer, 3.2 μl dNTPs (2.5 μM), 2.5 U hotstart Taq (Takara), and 2 μl DNA (10 μM). The PCR conditions were as follows: 95° for 3 min; 50 cycles of 95°C for 15 s, 56°C for 20 s, and 72°C for 30 s; and 72°C for 5 min. After extraction from the amplified product, single-stranded DNA was subjected to bisulfate modification using the EpiTect Bisulfite Kit (Qiagen) and pyrosequencing using the PyroMark Q96 ID System (Qiagen) with the primer 5'- TGGATGGGTAAAACCT-3'.

### DNA pyrosequencing for MGMT promoter methylation

Bisulfite modification of DNA was performed using the EpiTect Kit. The following primers were used to amplify the *MGMT* promoter region: (forward) 5'-GTTTYGGATATGTTGGGATA-3' and (reverse) 5'-biotin-ACCCAAACACTCACCAAATC-3'. PCR amplification was performed in a 40 μl reaction volume containing 0.5 μl each of the forward and reverse primers (10 μM), 4 μl 10× buffer, 3.2 μl dNTPs (2.5 μM), 2.5 U hotstart Taq, and 2 μl bisulfite-treated DNA (10 μM). The PCR conditions were as follows: 95°C for 3 min; 40 cycles of 95°C for 15 s, 52°C for 30 s, and 72°C for 30 s; and 72°C for 5 min. Single-stranded DNA was extracted from the amplified product using the QIAamp DNA Mini Kit and subjected to pyrosequencing using the PyroMark Q96 ID System with the primer 5'-GGATATGTTGGGATAGT-3' according to the manufacturer’s instructions. The methylation values acquired were averaged across the seven CpG loci tested within the *MGMT* promoter. LGG samples with an average methylation > 10% were considered *MGMT* promoter methylated.

### Detection of TP53 mutation and 1p/19q chromosome loss

Mutation scanning of *TP53* exons 5–8 was done using the following primers: 5'-AGGCCCTTAGCCTCTGTAAGC-3' (sense) and 5'-M13-CTGCTCAGATAGCGATGGTG-3' (antisense) for exon 5; 5'-M13-AGAAATCGGTAAGAGGTGGGC-3' (sense) and 5'-CATCCTGGCTAACGGTGAAAC-3' (antisense) for exon 6; 5'-M13-TTGGGCAGTGCTAGGAAAGAG-3' (sense) and 5'-GTTGGGAGTAGATGGAGCCTG-3' (antisense) for exon 7; 5'-TTGTCTTTGAGGCATCACTGC-3' (sense) and 5'-M13-GGAGCACTAAGCGAGGTAAGC-3' (antisense) for exon 8. PCR products were subsequently analyzed using Sanger sequencing. 1p/19q loss was detected by fluorescence *in situ* hybridization using LSI probe sets 1p36/1q25 and 19q13/19p13 (spectrum orange-labeled 1p36 and 19q13 probes; spectrum green-labeled 1q25 and 19p13 probes; Vysis) and evaluated in at least 200 non-overlapping nuclei with intact morphology.

### Statistical analysis

Progression-free survival (PFS) was calculated from the date of diagnosis to the date of recurrence or last follow-up. Overall survival (OS) was defined as the time from primary surgery to death. PFS and OS estimates were obtained using the Kaplan–Meier method and compared using the log-rank test. A p value ≤ 0.05 was considered significant. Cox proportional hazards regression was used to calculate the hazard ratios and 95% confidence intervals for the association of *IDH1* mutation, 1p/19q deletion, *TP53* mutation, *MGMT* promoter methylation, and clinical factors with prognosis.

## Results

### *IDH1* mutation status

Of the 417 grade II gliomas examined, mutations at codon 132 of the *IDH1* gene were detected in 309 tumors (74%) including 304 R132H mutations (arginine to histidine substitution [CGT to CAT]) and 5 R132G mutations (arginine to glycine substitution [CGT to GGT]), which resulted in amino acid sequence alterations.

### Association of IDH1 status with survival

**Fig 1:**
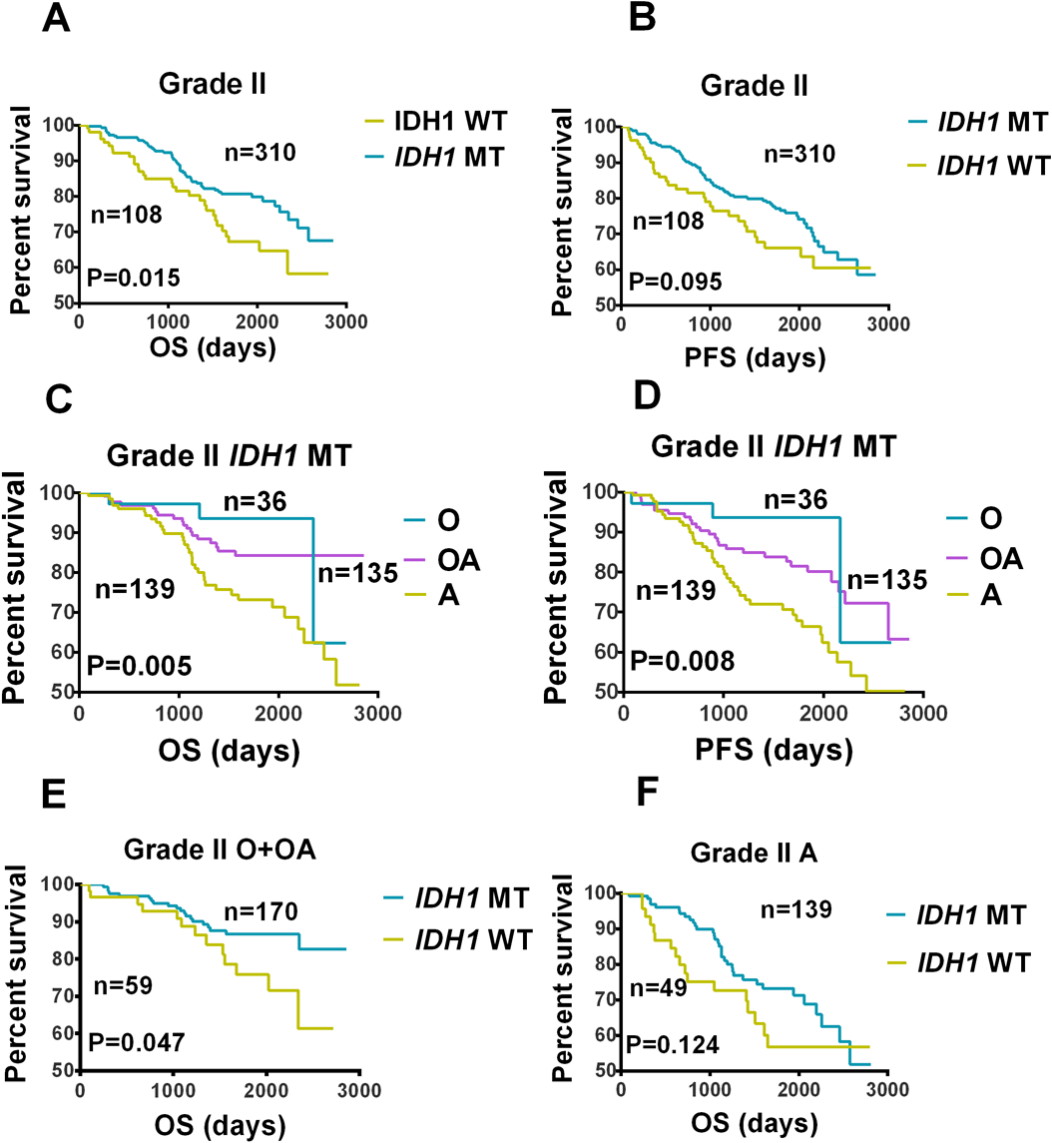
Kaplan-Meier Analysis of Overall Survival and Progression-free Survival in Patients with *IDH1*-mutated and Wild-type Gliomas. Comparison of (A) overall survival (OS) and (B) progression-free survival (PFS) between patients with *IDH1*-mutated (*IDH1* MT) and wild-type (*IDH1* WT) grade II gliomas. Comparison of (C) OS and (D) PFS among patients with *IDH1* MT grade II oligodendrogliomas (O), oliogoastrocytomas (OA), and atrocytomas (A). (E) A comparison of OS between patients with *IDH1* MT or *IDH1* WT grade II O or OA. (F) A comparison of OS between patients with *IDH1* MT or *IDH1* WT grade II As.

The study cohort consisted of 417 patients with grade II gliomas. Of the 417 patients, 309 and 108 patients had *IDH1*-mutated and wild-tumors, respectively. OS was significantly longer in patients with *IDH1*-mutated tumors than in patients with *IDH1* wild-type tumors (log-rank test, p = 0.015; Fig 1A). Although PFS was longer in patients with *IDH1*-mutated tumors than in patients with *IDH1* wild-type tumors, this difference was not significant (log-rank test, p = 0.095; Fig 1B). Histological subtype influenced the prognostic effect of *IDH1* mutation (log-rank test: OS p = 0.005, PFS p = 0.008; Fig 1C and 1D). *IDH1* mutation was associated with better OS in patients with oligoastrocytomas or oligodendrogliomas (p = 0.047; Fig 1E), but not in patients with astrocytomas (p = 0.124; Fig 1F).

The univariate regression analysis demonstrated that preoperative KPS score, chemotherapy, *TP53* mutation, 1p/19q loss, *MGMT* promoter methylation, and *IDH1* mutation were significantly associated with OS (Table 1), whereas preoperative KPS score, 1p/19q loss, and chemotherapy were associated with PFS (S1 Table). In the multivariate regression analysis, preoperative KPS score and *IDH1* mutation were identified as independent prognostic factors for OS (Table 1 and S3 Table), whereas only preoperative KPS score was shown to be an independent prognostic factor for PFS (S1 Table).

**Table 1:** Overall survival of grade II gliomas (n = 417).

Variables	Univariate analysis			Multivariate analysis		
	HR	95% CI	*P*-value	HR	95% Cl	*P*-value
**Gender**	0.825	0.529–1.287	0.397			
**Age**	1.012	0.990–1.035	0.290			
**Preoperative KPS**	0.968	0.952–0.984	<0.001	0.905	0.852–0.961	0.001
***IDH1*mutation**	0.607	0.384–0.958	0.032	0.253	0.087–0.735	0.012
***TP53* mutation**	1.676	1.028–2.735	0.039	1.989	0.684–5.788	0.207
**1p/19q loss**	0.596	0.357–0.996	0.048	0.675	0.171–2.666	0.575
**Chemotherapy**	2.515	1.409–4.489	0.002	0.723	0.266–1.967	0.526
***MGMT* promoter methylation**	1.039	0.385–2.804	0.939			
**Extent of resection**	0.932	0.684–1.271	0.656			
**Radiotherapy**	1.096	0.466–2.582	0.833			

### Association of IDH1 status with clinicopathological parameters

Next, we sought to ascertain the correlation of *IDH1* status with clinicopathological and molecular pathology features (Table 2). The *IDH1* mutation group consisted of 181 males and 128 females with a median age of 37 (range, 18–66) years, whereas the *IDH1* wild-type group consisted of 73 males and 34 females with a median age of 41 (range, 14–72) years. The median OS was 56 (range, 3–95) months and 49 (range, 3–93) months in the mutation and wild-type groups, respectively. *IDH1* mutation was associated with younger age (chi square test, p = 0.041). Median KPS score (chi square test, p = 0.200) and gender (chi square test, p = 0.078) were not significantly associated with *IDH1* status. Similarly, *IDH1* status did not differ according to histological subtype. A total of 224 (72%) and 64 (59%) *IDH1*-mutated and *IDH1* wild-type tumors were located in the frontal lobe, respectively (chi square test, p = 0.010). The Cancer Genome Atlas subtype was more favorable in the *IDH1* mutation group compared with the *IDH1* wild-type group (chi square test: proneural p = 0.001 and neural p = 0.022). Extent of resection was not significantly different between the two groups (chi square test, p = 0.246).

**Table 2:** Clinical and genetic features of WHO grade II gliomas (n = 417).

Characteristics	*IDH1* mutation (%)	*IDH1* wide-type (%)	*P*-value
**No. of cases**	309(74)	108(26)	
**Gender (F/M)**	128(41)/181	34(31)/73	0.078
**Age (<40 / ≥40yrs)**	187/125	52/55	0.041
**Histopathology**			
** Astrocytomas**	139(45)	49(45)	0.945
** Oligodendrogliomas**	36(12)	11(10)	0.678
** Oligoastrocytomas**	134(43)	48(44)	0.846
**Tumor location**			
** Frontal lobe**	224(72)	64(59)	0.010
** Temporal lobe**	105(34)	45(42)	0.152
** Parietal lobe**	23(7)	8(7)	0.288
** Insula**	63(20)	23(21)	0.841
** Occipital lobe**	6(2)	6(6)	0.110
** Others **	20(6)	12(11)	0.119
**Extent of resection**			
** GTR (%)**	109(38)/176	44(45)/54	0.246
**Molecular biomarkers**			
** *MGMT* promoter methylation (high/low)**	42(65)/23	6(33)/12	0.017
** 1p*/*19q loss (Yes/No)**	89(30)/208	32(31)/71	0.834
** Mutant *TP53* (Yes/No)**	81(36)/145	7(9)/73	<0.001

### Molecular features

**Fig 2:**
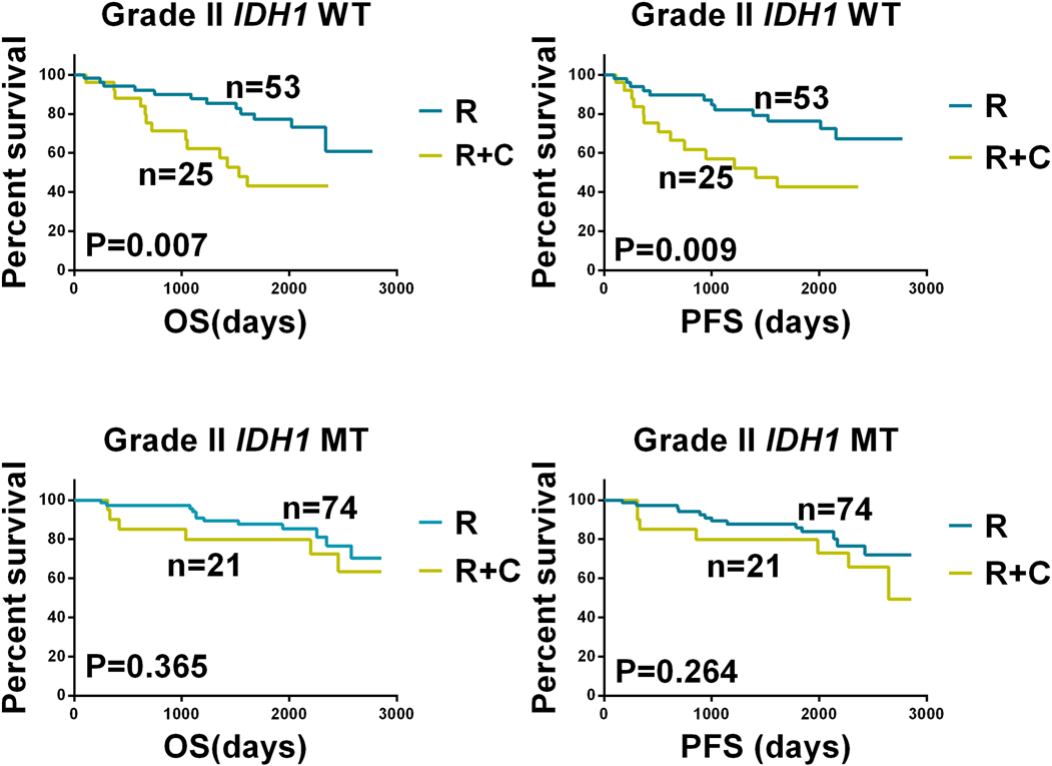
Kaplan-Meier Analysis of Overall Survival and Progression-free Survival According to *IDH1* Status and Adjuvant Treatment. A comparison of (A) overall survival (OS) and (B) progression-free survival (PFS) between patients with *IDH1* wild-type (*IDH1* WT) grade II gliomas who received radiotherapy (RT) or radiotherapy plus chemotherapy (RT+CT). A comparison of (C) OS and (D) PFS between patients with *IDH1*-mutated (*IDH1* MT) grade II gliomas who received RT or RT+CT. For the *IDH1* WT group, PFS and OS were longer in patients who underwent RT compared with those who underwent RT+CT (p = 0.002). For the *IDH1* MT group, PFS and OS were not significantly different between patients who underwent RT or RT+CT (p = 0.194).

*MGMT* promoter methylation status could be determined in 83 tumors. *MGMT* promoter methylation was present in 48 (58%) tumors (Table 2). Strikingly, of the 48 tumors with *MGMT* promoter methylation, 42 (87%) were *IDH1*-mutated tumors, whereas only 6 (13%) were *IDH1* wild-type tumors (chi square test, p = 0.017). The proportion of patients with *TP53* mutations was higher in the *IDH1* mutation group than in the *IDH1* wild-type group (36% [81/226] vs. 9% [7/80]; chi square test, p < 0.001). The frequency of 1p/19q deletion was not significantly different between the *IDH1* mutation and wild-type groups (30% vs. 31%; chi square test, p = 0.834). Furthermore, in the *IDH1* wild-type group, OS and PFS were significantly longer in patients who underwent radiotherapy than those who underwent radiotherapy plus chemotherapy (p = 0.002 and p = 0.003, respectively). In contrast, OS and PFS were not significantly different between patients who received radiotherapy or radiotherapy plus chemotherapy in the *IDH1* mutation group (p = 0.194 and p = 0.137, respectively; Fig 2).

## Discussion

Using large-scale sequencing, nearly 12% of GBMs were found to harbor *IDH1* mutations [13]. Approximately 70% of *IDH1* mutations were found in grade II and III gliomas and secondary GBMs [5]. Yan et al.[3] reported that more than 70% of WHO grade II and III astrocytomas and oligodendrogliomas had mutations in amino acid position 132 of *IDH1*. *IDH1* mutations also have been shown to occur predominantly in younger patients [5]. Several previous studies have demonstrated the important prognostic role of *IDH1* mutations in patients with high-grade gliomas [12, 14]. Sanson et al. [15] reported that *IDH1* mutation was associated with longer survival in the univariate and multivariate analyses of 404 gliomas, including 100 grade II gliomas [15]. A study of 139 LGGs demonstrated an association between *IDH1* mutation and OS [16]. In contrast, a study with a larger sample size (n = 360) suggested that *IDH1* mutation does not have prognostic value in LGGs [17]. Therefore, the predictive role of *IDH1* mutation in WHO grade II gliomas is controversial and remains to be established. We investigated the frequency and prognostic impact of *IDH1* mutations in a large LGG dataset. Our results demonstrated that *IDH1* mutations occur at a high frequency in WHO grade II astrocytomas and oligodendrogliomas. Furthermore, *IDH1* mutation was found to be a robust predictor of patient survival, which corroborates prior reports [8, 15, 16].

1p/19q loss, *TP53* mutation, and *MGMT* promoter methylation have been investigated as potential prognostic predictors in glioma. Most previous studies have suggested that OS is longer in LGG patients with combined 1p and 19q loss [18]. However, the prognostic role of *TP53* mutations has remained controversial, and no consistent association of *TP53* mutation with survival outcome has been reported. In a cohort study of 159 patients with LGGs, PFS, but not OS, was significantly shorter in patients with *TP53*-mutated tumors than in those with *TP53* wild-type tumors [19]. Another study of 122 glioma cases demonstrated that *TP53* mutation is a predictor of shorter survival in patients with low-grade diffuse gliomas [20]. Moreover, evaluation of the prognostic role of *MGMT* promoter methylation is complicated by the influence of clinical factors, such as age and histopathology [21]. Our study underscores the prognostic relevance of 1p/19q loss and *TP53* mutation, but not *MGMT* promoter methylation, in LGGs [16]. Notably, our study is the first to demonstrate the prognostic significance of *IDH1* mutation in patients with oligodendroglioma or oligoastrocytoma, but not in patients with astrocytoma.

Several clinical parameters and genetic factors have been shown to be related to *IDH1* status. Parsons et al. [13] found that mean age was significantly lower in *IDH1*-mutated GBMs than in those with *IDH1* wild-type GBMs. In the present study, we showed that patients with LGGs carrying *IDH1* mutations were significantly younger than those with *IDH1* wild-type LGGs. In line with previous reports, we found that *IDH1* mutation is correlated with *MGMT* promoter methylation. A high level of *MGMT* promoter methylation was detected in 65% of patients in the *IDH1* mutation group, whereas only 33% of patients in the *IDH1* wild-type group had a high level of *MGMT* promoter methylation. This finding indicates that *MGMT* hypermethylation is highly associated with *IDH1* mutation. Furthermore, Watanabe et al. [5] demonstrated the copresence of *IDH1* and *TP53* mutations in 63% of low-grade astrocytomas, whereas *IDH1* mutation plus 1p/19q loss was present in most (64%) oligodendrogliomas. In concordance with previous reports [3, 22], we observed a highly significant correlation between *TP53* mutation and *IDH1* mutation. Interestingly, the frequency of 1p/19q loss was not significantly different between the *IDH1* mutation and wild-type groups. To date, few studies have focused on the association between tumor location and *IDH1* mutation. Yan et al. [12] and Zhang et al. [14] reported that *IDH1*-mutated primary GBMs and anaplastic astrocytomas mainly involved the frontal lobe. Similarly, we found that a higher proportion of *IDH1*-mutated LGGs were located in the frontal lobe compared with wild-type LGGs.

GBM patients with *MGMT* promoter methylation have been reported to have a higher response rate to temozolomide [23]; however, whether *IDH1* mutation can predict outcome to this specific treatment is unknown. Previously, a Dutch study [24] revealed that *IDH1* mutation was a good predictor of OS but not response to temozolomide after radiotherapy in patients with low-grade astrocytomas, which is in agreement with our findings. Furthermore, in the *IDH1* mutation group, we found that survival outcome was not different between patients who did and did not receive chemotherapy (primarily temozolomide). However, in the wild-type *IDH1* group, patients who underwent radiotherapy plus chemotherapy had a shorter survival than those who only underwent radiotherapy. Despite the wide clinical application of chemotherapy in GBM patients, our results suggest that chemotherapy is unsuitable for patients without *IDH1* mutations. This may be because the toxic effects of temozolomide are even more apparent in LGGs. Our study suggests that wild-type *IDH1* status is associated with good response to radiotherapy but not to radiotherapy plus chemotherapy. This association and its underlying mechanisms require further investigation.

## Conclusions

*IDH1* mutation was an independent prognostic factor in LGGs; however, it had no predictive value for chemotherapy response. *IDH1* mutation was highly associated with *MGMT* promoter methylation and *TP53* mutation.

## Supporting Information

S1 Table Progression-free Survival of 417 Patients with Grade II Gliomas. (DOCX)

S2 Table The DOIs of the WHO Grade II Glioma Cases Used in this Study. (DOCX)

S3 Table Multivariate Analysis for Overall Survival (n = 417). (DOCX)
