# Supplementary material for: Isocitrate dehydrogenase 1 Gene Mutation Is Associated with Prognosis in Clinical Low-Grade Gliomas
Source: PLoS One. 2015 Jun 26;10(6):e0130872. doi: 10.1371/journal.pone.0130872 (PMC4482584; doi:10.1371/journal.pone.0130872)
Supplement: S1 Table — (DOCX) [file pone.0130872.s001.docx]

**Table S1. Progression-free survival of grade II gliomas (n = 417)**

|  | **Univariate analysis** | | |  | **Multivariate analysis** | | |
| --- | --- | --- | --- | --- | --- | --- | --- |
| **Variables** | **HR** | **95% CI** | ***P*-value** |  | **HR** | **95% CI** | ***P*-value** |
| **Gender** | 0.929 | 0.620-1.391 | 0.720 |  |  |  |  |
| **Age** | 1.017 | 0.997-1.038 | 0.104 |  |  |  |  |
| **Preoperative KPS** | 0.971 | 0.956-0.987 | <0.001 |  | 0.933 | 0.891-0.976 | <0.001 |
| **Extent of resection** | 1.037 | 0.677-1.589 | 0.867 |  |  |  |  |
| ***IDH1* mutation** | 0.708 | 0.458-1.095 | 0.121 |  |  |  |  |
| ***MGMT* promoter methylation** | 1.090 | 0.404-2.943 | 0.865 |  |  |  |  |
| ***TP53* mutation** | 1.489 | 0.939-2.363 | 0.091 |  |  |  |  |
| **1p/19q loss** | 0.566 | 0.352-0.912 | 0.019 |  | 0.455 | 0.148-1.402 | 0.170 |
| **Chemotherapy** | 2.459 | 1.417-4.265 | 0.001 |  | 0.889 | 0.366-2.159 | 0.795 |
| **Radiotherapy** | 0.865 | 0.407-1.836 | 0.705 |  |  |  |  |
