# Supplementary material for: Isocitrate dehydrogenase 1 Gene Mutation Is Associated with Prognosis in Clinical Low-Grade Gliomas
Source: PLoS One. 2015 Jun 26;10(6):e0130872. doi: 10.1371/journal.pone.0130872 (PMC4482584; doi:10.1371/journal.pone.0130872)
Supplement: S2 Table — (DOCX) [file pone.0130872.s002.docx]

**Table S2. The DOIs of WHO grade II glioma cases used in this study**

| **Sample ID (DOI*)** | **Gender** | **Age** | **OS** | **Censor** |
| --- | --- | --- | --- | --- |
| **323** | **female** | **49** | **2351** | **0** |
| **131** | **male** | **59** | **109** | **1** |
| **313** | **male** | **32** | **2363** | **0** |
| **73** | **female** | **44** | **240** | **1** |
| **369** | **male** | **33** | **2298** | **0** |
| **821** | **female** | **41** | **1339** | **0** |
| **258** | **female** | **61** | **663** | **1** |
| **703** | **female** | **31** | **1355** | **1** |
| **814** | **male** | **60** | **1162** | **0** |
| **746** | **male** | **45** | **332** | **1** |
| **37** | **male** | **72** | **2799** | **0** |
| **45** | **female** | **56** | **239** | **1** |
| **781** | **male** | **36** | **1721** | **0** |
| **250** | **male** | **51** | **586** | **0** |
| **574** | **male** | **51** | **1415** | **0** |
| **217** | **male** | **45** | **1341** | **0** |
| **266** | **male** | **38** | **2444** | **0** |
| **766** | **male** | **32** | **1735** | **0** |
| **832** | **male** | **30** | **1152** | **0** |
| **550** | **male** | **38** | **1088** | **1** |
| **805** | **male** | **46** | **1675** | **0** |
| **41** | **male** | **53** | **94** | **1** |
| **844** | **male** | **33** | **1142** | **0** |
| **421** | **male** | **44** | **2227** | **0** |
| **112** | **male** | **18** | **1533** | **1** |
| **385** | **female** | **41** | **1039** | **1** |
| **450** | **male** | **26** | **2199** | **0** |
| **42** | **male** | **39** | **1917** | **0** |
| **790** | **male** | **47** | **1198** | **0** |
| **269** | **female** | **45** | **132** | **0** |
| **59** | **male** | **35** | **2772** | **0** |
| **475** | **male** | **14** | **2078** | **0** |
| **627** | **female** | **35** | **1898** | **0** |
| **619** | **male** | **41** | **564** | **1** |
| **493** | **female** | **33** | **2056** | **0** |
| **185** | **male** | **38** | **372** | **1** |
| **328** | **female** | **21** | **1610** | **1** |
| **784** | **male** | **47** | **587** | **0** |
| **297** | **female** | **31** | **2393** | **0** |
| **659** | **female** | **51** | **730** | **0** |
| **291** | **female** | **51** | **1736** | **0** |
| **347** | **female** | **27** | **2338** | **0** |
| **99** | **male** | **30** | **2715** | **0** |
| **422** | **male** | **47** | **2225** | **0** |
| **691** | **male** | **42** | **1826** | **0** |
| **542** | **male** | **25** | **887** | **0** |
| **418** | **male** | **46** | **2228** | **0** |
| **552** | **female** | **37** | **1987** | **0** |
| **626** | **male** | **21** | **1898** | **0** |
| **688** | **male** | **19** | **1520** | **0** |
| **514** | **female** | **53** | **2022** | **1** |
| **440** | **male** | **45** | **2187** | **0** |
| **828** | **male** | **43** | **1155** | **0** |
| **125** | **male** | **20** | **277** | **1** |
| **566** | **male** | **41** | **1653** | **1** |
| **173** | **male** | **29** | **2596** | **0** |
| **846** | **female** | **39** | **1057** | **0** |
| **201** | **female** | **25** | **1411** | **1** |
| **770** | **male** | **31** | **1422** | **0** |
| **424** | **male** | **27** | **2220** | **0** |
| **520** | **male** | **24** | **2022** | **0** |
| **559** | **female** | **44** | **746** | **1** |
| **863** | **female** | **43** | **1118** | **0** |
| **705** | **male** | **41** | **1507** | **1** |
| **807** | **male** | **43** | **1692** | **0** |
| **739** | **male** | **46** | **1051** | **1** |
| **271** | **male** | **42** | **1426** | **1** |
| **204** | **male** | **60** | **618** | **1** |
| **219** | **male** | **33** | **379** | **1** |
| **321** | **male** | **42** | **1681** | **0** |
| **392** | **male** | **17** | **184** | **0** |
| **276** | **male** | **44** | **529** | **0** |
| **638** | **male** | **42** | **1881** | **0** |
| **871** | **male** | **37** | **1107** | **0** |
| **633** | **female** | **57** | **1890** | **0** |
| **888** | **male** | **30** | **669** | **1** |
| **706** | **male** | **36** | **619** | **1** |
| **610** | **female** | **25** | **1918** | **0** |
| **296** | **male** | **48** | **1215** | **0** |
| **395** | **male** | **54** | **1551** | **1** |
| **118** | **female** | **27** | **512** | **0** |
| **294** | **male** | **54** | **197** | **0** |
| **398** | **male** | **28** | **2256** | **0** |
| **786** | **male** | **59** | **1234** | **1** |
| **455** | **female** | **47** | **2178** | **0** |
| **268** | **female** | **18** | **2438** | **0** |
| **482** | **male** | **46** | **2070** | **0** |
| **500** | **female** | **36** | **1520** | **0** |
| **763** | **male** | **52** | **317** | **0** |
| **668** | **male** | **28** | **1677** | **1** |
| **143** | **female** | **36** | **1056** | **1** |
| **362** | **female** | **39** | **1680** | **0** |
| **15** | **male** | **32** | **1972** | **0** |
| **787** | **male** | **38** | **1709** | **0** |
| **411** | **male** | **36** | **2245** | **0** |
| **662** | **female** | **59** | **300** | **1** |
| **117** | **female** | **34** | **2689** | **0** |
| **771** | **male** | **59** | **154** | **0** |
| **267** | **male** | **37** | **1374** | **1** |
| **900** | **male** | **38** | **1075** | **0** |
| **883** | **female** | **39** | **1016** | **0** |
| **363** | **male** | **43** | **130** | **0** |
| **394** | **male** | **45** | **1899** | **0** |
| **39** | **male** | **26** | **2799** | **0** |
| **452** | **male** | **30** | **1815** | **0** |
| **529** | **male** | **35** | **2014** | **0** |
| **445** | **male** | **35** | **2063** | **1** |
| **712** | **male** | **25** | **1111** | **0** |
| **548** | **male** | **39** | **875** | **0** |
| **556** | **male** | **37** | **1980** | **0** |
| **723** | **female** | **25** | **1768** | **0** |
| **91** | **male** | **42** | **231** | **0** |
| **737** | **male** | **36** | **1133** | **1** |
| **D04** | **male** | **37** | **1351** | **0** |
| **D20** | **female** | **40** | **1316** | **0** |
| **106** | **male** | **30** | **2708** | **0** |
| **376** | **male** | **30** | **108** | **0** |
| **862** | **male** | **31** | **1046** | **0** |
| **326** | **male** | **45** | **393** | **1** |
| **456** | **male** | **44** | **1804** | **0** |
| **246** | **male** | **46** | **332** | **1** |
| **D19** | **female** | **64** | **297** | **1** |
| **538** | **female** | **43** | **1451** | **0** |
| **812** | **male** | **27** | **1163** | **0** |
| **618** | **female** | **32** | **1375** | **1** |
| **315** | **female** | **33** | **2380** | **0** |
| **909** | **female** | **40** | **1065** | **0** |
| **361** | **male** | **55** | **167** | **0** |
| **536** | **female** | **33** | **2077** | **0** |
| **183** | **female** | **47** | **1566** | **1** |
| **435** | **male** | **31** | **1320** | **1** |
| **320** | **male** | **32** | **2295** | **0** |
| **480** | **female** | **46** | **314** | **0** |
| **468** | **female** | **65** | **2158** | **0** |
| **428** | **female** | **42** | **1189** | **1** |
| **1031** | **female** | **33** | **415** | **0** |
| **595** | **female** | **52** | **1390** | **0** |
| **1368** | **male** | **49** | **301** | **0** |
| **1369** | **female** | **37** | **360** | **0** |
| **625** | **female** | **34** | **1903** | **0** |
| **282** | **male** | **44** | **247** | **1** |
| **76** | **male** | **37** | **662** | **1** |
| **134** | **male** | **53** | **1208** | **1** |
| **621** | **male** | **41** | **1347** | **0** |
| **479** | **female** | **37** | **2072** | **0** |
| **77** | **female** | **50** | **860** | **0** |
| **469** | **male** | **31** | **2161** | **0** |
| **898** | **male** | **38** | **441** | **0** |
| **36** | **female** | **18** | **300** | **1** |
| **61** | **male** | **49** | **2771** | **0** |
| **16** | **female** | **42** | **1924** | **0** |
| **897** | **female** | **39** | **1080** | **0** |
| **528** | **female** | **52** | **2014** | **0** |
| **720** | **female** | **46** | **1258** | **1** |
| **388** | **male** | **49** | **2276** | **0** |
| **53** | **female** | **51** | **1901** | **0** |
| **316** | **female** | **31** | **1689** | **0** |
| **905** | **male** | **46** | **1068** | **0** |
| **502** | **male** | **40** | **1489** | **0** |
| **776** | **female** | **48** | **1724** | **0** |
| **531** | **female** | **42** | **2009** | **0** |
| **261** | **male** | **28** | **2458** | **0** |
| **809** | **male** | **26** | **1252** | **1** |
| **711** | **male** | **50** | **1798** | **0** |
| **698** | **female** | **62** | **1798** | **0** |
| **19** | **male** | **24** | **1527** | **1** |
| **50** | **male** | **25** | **1353** | **0** |
| **D12** | **female** | **28** | **1329** | **0** |
| **54** | **male** | **32** | **2776** | **0** |
| **20** | **male** | **34** | **1947** | **0** |
| **6** | **male** | **36** | **1983** | **0** |
| **72** | **male** | **41** | **623** | **0** |
| **86** | **male** | **42** | **2578** | **1** |
| **670** | **female** | **36** | **1841** | **0** |
| **823** | **female** | **52** | **656** | **1** |
| **751** | **male** | **35** | **1750** | **0** |
| **543** | **male** | **43** | **1995** | **0** |
| **856** | **male** | **54** | **1297** | **0** |
| **283** | **female** | **48** | **2410** | **0** |
| **511** | **male** | **32** | **2030** | **0** |

*DOIs are patients’ ID used in the Chinese Giloma Genome Atlas

**Annotation**

All clinical information of the cases collected by the Chinese Giloma Genome Atlas could be accessed by applying at the website (http://www.cgga.org.cn/). All information of the data could only be used for scientific research.
