# Supplementary material for: Isocitrate dehydrogenase 1 Gene Mutation Is Associated with Prognosis in Clinical Low-Grade Gliomas
Source: PLoS One. 2015 Jun 26;10(6):e0130872. doi: 10.1371/journal.pone.0130872 (PMC4482584; doi:10.1371/journal.pone.0130872)
Supplement: S3 Table — (DOCX) [file pone.0130872.s003.docx]

**S3 Table. Multivariate Analysis for Overall Survival (n = 417).**

| **Variables** |  | **HR** | **95% Cl** | ***P*-value** |
| --- | --- | --- | --- | --- |
| **Age (≥ 40 years)** |  | 0.980 | 0.943−1.018 | 0.299 |
| **Preoperative KPS** |  | 0.929 | 0.900−0.958 | <0.001 |
| ***IDH1* mutation** |  | 0.308 | 0.108−0.874 | 0.027 |
| ***TP53* mutation** |  | 2.011 | 0.710−5.697 | 0.189 |
| **1p/19q loss** |  | 0.581 | 0.156−2.159 | 0.417 |
| **Chemotherapy** |  | 0.973 | 0.373−2.540 | 0.955 |
| **Extent of resection** |  | 0.763 | 0.308−1.888 | 0.558 |

CI, confidence interval; HR, hazard ratio; IDH1, isocitrate dehydrogenase 1; KPS, Karnofsky Performance Status.
